# 

**Published:** 1862-05

**Authors:** 


### AMERICAN DENTAL ASSOCIATION.

This body meets in Cleveland on the last Tuesday of July next, and we hope will be fully attended. The work which this Association professes to do is not and has not been done by any other. It is as we conceive the elaboration and development of the *science* of the profession,—that upon which true practice is based.

The art of the profession would of course come within range of this Association, but it would certainly be secondary to the consideration of foundation principles. There are some things, perhaps, that have been matters of controversy, that could be profitably discussed by this Association; it would not be well, however, that any definite action should be taken upon any such question, at least any that would be considered binding.

Let the influence be wholly such as would arise from the proper presentation of truth: the most that should be attempted in this direction would be suggestive or recommendatory.

This is a delegated body, yet all may profit by its labors, and in many ways coöperate.

We hope to see all who have the good of our profession at heart present at that meeting. T.

### DELAY.

This No. of the Register is much behind in the time of issue. This is so for two reasons,—we had not the copy for the original department till after it should have been issued; and again, our time during the latter part of April and the fore part of May was so entirely occupied by things that we could not control, that it left no time for this work. We hope there will be no such interruption in future. T.

BUFFALO, N. Y., May 10th, 1862.

THE AMERICAN DENTAL CONVENTION will hold its Eighth Annual Session at Trenton Falls, N. Y., commencing on Tuesday, the fifth day of August next, at 10 o'clock A.M. You are respectfully and cordially invited to attend.

The Executive Committee reported, at the last Annual Convention, the following order of business, and subjects for discussion, for the next Convention :

ORDER OF BUSINESS.

1st. Admission of Members.
2d. Reading Minutes of the last Convention.
3d. Report of Officers and Committees.
4th. Election of Officers.
5th. Retiring President's Address.
6th. Induction of Officers.

All essays shall be read to open the discussion on the subjects to which they relate.

No member shall speak more than ten minutes, nor more than twice on the same subject without permission.

I. *Miscellaneous Subjects.*—1. Anæsthetics. Their use and relative value. 2. Alveolar abscess. 3. The causes influencing an abnormal development of the teeth.

II. *Operative Dentistry.*—1. Filling Teeth. Simple and complicated cavities. 2. The Dental pulp. Its varied treatment. 3. The extraction of teeth.

III. *Mechanical Dentistry.*—1. Artificial Dentures. Temporary and Permanent.

IV. *Unfinished Business.*

N. B.—The Executive Committee suggest that half an hour every morning be devoted to the presentation of models, improvements and inventions, and the disposal of business not embodied in the regular order.

Signed,

W. H. ATKINSON,

G. T. BARKER,

W. B. ROBERTS,

I. J. WETHERBEE,

SAMUEL MALLET,

} *Executive Committee.*

This is a CONVENTION where all regular practicing DENTISTS

may unite in the free discussion of subjects relating to, and the full interchange of opinions, experience and practice of the Science of Dentistry. As associated action is the surest means for advancement, it is hoped that by personal attendance, the contribution of essays, and discussion of the subjects which have been presented by the Executive Committee, you will add to the interest of the Convention, and the elevation and advancement of the profession of Dental Surgery.

Yours, Very Respectfully,
B. T. WHITNEY.

The American Dental Association will meet in the City of Cleveland, Ohio, upon the last Tuesday of July next, at ten o'clock A. M. W. Muir Rogers,
*Shelbyville, Ky.*, *Corresponding Sec'y.*
May 26, 1862.

Committees are to report at that meeting upon the following subjects, viz.:

1. *Dental Physiology.*
2. *Dental Pathology and Surgery.*
3. *Mechanical Dentistry.*
4. *Dental Education.*
5. *Dental Literature.*
6. *Dental Chemistry.*

Upon each of these we expect full and interesting reports will be presented. Ed.

### DENTAL REGISTER,

### OF THE WEST.

Issued Monthly, at $3 a year in advance.

DEVOTED TO THE INTERESTS OF THE DENTAL PROFESSION.

**Edited by J. TAFT and GEO. WATT.**

The Volume commences in January and closes in December.

The Register being issued monthly is always fresh, therefore indispensable to the practitioner who desires to keep posted up with the new inventions and improvements which are daily developed. Neither expense nor effort will be spared to give value and variety to its contents. Articles will be illustrated with well executed engravings, whenever they will add to the interest or assist in explanation.

Its extensive circulation and frequency of issue makes it the BEST DENTAL ADVERTISING MEDIUM in the United States.

☞CONTRIBUTIONS SOLICITED.

Address Original Communications to **Geo. Watt, Xenia, O.**

Exchanges to **J. Taft**, or **Dental Register, Cincinnati, Ohio.**

Business Communications to

J. TAFT, Publisher,
56 West Fourth Street, Cincinnati, O.

JNO. T. TOLAND, Proprietor,
38 West Fourth Street, Cincinnati, O.

Communications must reach the Editor as early as the 15th, to insure insertion in the next issue.

